# A Combined RNA Preservation and Extraction Protocol for Gene Expression Studies in Cacao Beans

**DOI:** 10.3389/fpls.2020.00992

**Published:** 2020-06-30

**Authors:** Jocelyn De Wever, Dieter Tulkens, Jan Verwaeren, Helena Everaert, Hayley Rottiers, Koen Dewettinck, Steve Lefever, Kathy Messens

**Affiliations:** ^1^ Research Unit Molecular Biotechnology, Department of Biotechnology, Faculty of Bioscience Engineering, Ghent University, Ghent, Belgium; ^2^ Center for Medical Genetics Ghent (CMGG), Ghent University Hospital, Ghent, Belgium; ^3^ Research Unit Knowledge-based Systems (KERMIT), Department of Data Analysis and Mathematical Modelling, Faculty of Bioscience Engineering, Ghent University, Ghent, Belgium; ^4^ Food Structure & Function Research Group (FSF), Department of Food Technology, Safety and Health, Faculty of Bioscience Engineering, Ghent University, Ghent, Belgium; ^5^ Cancer Research Institute Ghent (CRIG), Ghent University, Ghent, Belgium; ^6^ Bioinformatics Institute Ghent (BIG), Ghent University, Ghent, Belgium

**Keywords:** RNA, *Theobroma cacao* L., extraction, freeze-drying, RNA quality numbers, preservation, transportation, cacao bean

## Abstract

Despite the high economic importance of cacao beans, few RNA-based studies have been conducted on this plant material and hence no optimal RNA-extraction has been reported. Moreover, extraction of high-quality RNA from recalcitrant cacao bean tissue has shown many difficulties and requires optimization. Furthermore, cacao beans are mostly found at remote and under-resourced locations, which pressures the outsourcing of such analysis and thereby demands RNA-stable preservation and transportation of cacao beans. This study aims to select an appropriate RNA extraction and preservation/transportation method for cacao beans. For this purpose, three sample homogenization and five extraction protocols on cacao beans were compared. In addition, 13 preservation conditions—differing in tissue crushing degree, preservation method, duration, and temperature—were compared and evaluated. A comparative analysis revealed that CTAB-based homogenization and extraction outcompeted all tested commercial protocols in RNA yield and integrity, respectively. Preservation at −80°C affected RNA quality the least, whereas freeze-drying was most suitable for transportation at room temperature for maximum 1 week. The cacao bean RNA obtained from the selected methods were compatible for downstream applications. The results of this study will facilitate on-field sampling and transportation of genetically sensitive cacao material prior to cacao bean transcriptomic studies. In addition, valuable insights on sample homogenization, extraction, preservation, and transportation have been provided, which is of interest to every plant geneticist.

## Introduction

The beans of the cacao tree (*Theobroma cacao* L.) are of high economic importance since they are the raw material for cacao liquor and chocolate (Guiltinan et al., 2008). Few transcriptomic-based studies have been conducted on the cacao beans, although these could help to unravel the genetic background underlying its highly desired flavor (Argout et al., 2008; Argout et al., 2011). A prerequisite to conduct such research is to obtain high-quality RNA (Bustin et al., 2009; Gallego Romero et al., 2014). Since RNA is highly sensitive to degradation, special attention should be paid to preservation and transportation of the cacao beans and the efficiency of the used extraction methods at the level of yield, quality, and integrity (Sangha et al., 2010; Fabre et al., 2014; Gallego Romero et al., 2014).

The isolation of sufficient high-quality RNA from cacao material and other plant-related tissues has shown complicated, due to the presence of interfering metabolites (Gesteira et al., 2003; Rodrigues et al., 2007; Varma et al., 2007; Pereira et al., 2017). Further, a well-defined, cross-tissue compatible RNA extraction protocol is not yet available and several attempts to extract high-quality and intact RNA from complex plant tissues have failed when using standard extraction protocols and kits (Sangha et al., 2010; Fabre et al., 2014; Royaert et al., 2016; Silva et al., 2016). Hence, every plant geneticist should optimize a suitable RNA-extraction protocol specific for the tissue under study. To date, a wide variety of strategies to optimize the maceration, preparation, and extraction of high-quality RNA from difficult plant tissues has been reported. Examples include varying extraction conditions (kits *versus* manual protocols) and sample homogenization procedures (chemical *versus* mechanical) (Portillo et al., 2006; Rodrigues et al., 2007; Vomelová et al., 2009; Gudenschwager et al., 2012; Kalinowska et al., 2012).

Current cacao-related extraction workflows mostly focus on leaf RNA (Gesteira et al., 2003; Leal et al., 2007; Bailey et al., 2014; Legavre et al., 2015; Fister et al., 2016), using either 3% CTAB based protocols or commercially available RNAqueous (Bertolde et al., 2014) and RNeasy^®^ Plant Mini (Gesteira et al., 2007) kits. The first RNA extraction from cacao beans was described in 1991 (Spencer and Hodge, 1991) and was successfully applied in 1992 for the identification of certain polypeptides (Spencer and Hodge, 1992). In 2002, Jones *et al.* extracted total RNA from cacao beans using two kit-independent protocols. However, to the best of our knowledge, no state-of-the-art comparison of different cacao bean RNA extraction techniques—focused on RNA quality and quantity—has been reported so far.

Cacao is mainly cultivated in developing countries with limited resources for optimal RNA extraction and analysis. Therefore, RNA extraction and analyses—following (overseas) shipment—is often the only feasible option. Consequently, preservation methods compatible with long-term transportation should be considered. The gold standard for RNA preservation recommends cryopreservation of the tissue at −80°C or in liquid nitrogen (Burden, 2008; García-baldenegro et al., 2015). Unfortunately, transporting samples at ultra-low preservation temperatures has been shown difficult as most shipping companies (especially air-based) reject samples stored on dry ice or in liquid nitrogen (Hernandez et al., 2009; García-baldenegro et al., 2015). Therefore, alternative preservation methods for cacao beans should be evaluated. The samples could be transported at suboptimal cooling conditions, such as −20 or 4°C. Although this is highly discouraged as freeze-thaw cycles promote degradation of the RNA, the effects of suboptimal cooling on in-tissue preserved RNA have not yet been equivocally established. An alternative is freeze drying (FD), which has been considered as a cost-effective method for temporary RNA preservation and short-term shipment of genetic sensitive samples at room temperature (RT). This method has already been successfully applied on, for instance, grapes (García-baldenegro et al., 2015), bananas (Lassois et al., 2009), and tea leaves (Jaiprakash et al., 2003).

**Figure 1:**
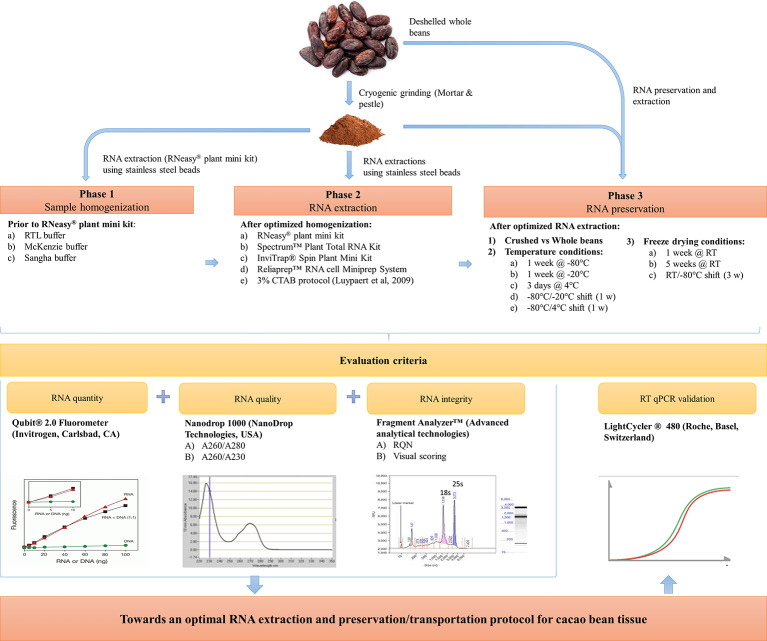
Schematic overview towards an efficient sample homogenization, RNA extraction, and preservation method for cacao bean tissue.

This work is divided into three phases and offers a detailed comparison of several sample homogenization, extraction, and preservation methods compatible with overseas transportation and wet-lab processing specifically on cacao beans, but of interest to every plant geneticist. For this purpose, three homogenization workflows and five RNA extraction protocols have been evaluated on cacao beans in phases 1 and 2, respectively (**Figure 1**). The most efficient RNA extraction method in terms of ease-of-use, RNA quality and quantity in phase 2, was selected to assess the impact of various preservation/transportation conditions on RNA quantity and integrity in phase 3. Finally, the quality of the obtained cacao bean RNA from the most suitable extraction and preservation workflow was evaluated independently with RT-qPCR.

## Methods

### Plant Material

Five mature cacao pods from the R1/257 cultivar were sampled at the Cocoa Research Institute Ghana (CRIG) and transported in a cardboard box to Belgium with DHL. Ten days after collection, pods arrived in-house and were immediately opened. Seeds were removed from the pulp and their seed coat. The beans were partially preserved as a whole (W), while the remainder was cryogenically crushed (C) using a precooled mortar and pestle. Both tissue forms were preserved in sealed eppendorfs or bags at −80°C prior to extraction.

For independent validation of the selected extraction and preservation method with RT-qPCR, six cacao beans from the CCN 51 variety were sampled on-field in Ecuador (Joya de los Sachas). A mature pod was harvested, opened, and beans were preserved according to optimized methods (see *Method Validation With RT-qPCR*).

### Phase 1: Sample Homogenization Workflows

Three homogenization workflows, based on three different lysis buffers, were compared in combination with the RNeasy^®^ Plant Mini Kit (Qiagen, Inc., Chatsworth, CA). The used buffers were either (A) kit-included (RTL), (B) guanidine thiocyanate-based (MacKenzie et al., 1997), or (C) CTAB-based (Sangha et al., 2010) (**Figure 1**). The latter two will be referred to as MacKenzie and Sangha buffer, respectively. To increase extraction efficiency, stainless steel beads were used during each homogenization.

The first homogenization workflow (A) was performed as described by the manufacturer (Qiagen, Inc., Chatsworth, CA). In the second workflow (B), 500 µl of MacKenzie buffer, *i.e.* [4M guanidine thiocyanate, 0.2 M sodium acetate (pH 5.0), 25 mM ethylenediaminetetraacetic acid (EDTA), 2.5% (w/v) poly-vinyl pyrrolidone (PVP), and 1% (v/v) β-mercaptoethanol], was added to 100 mg of crushed cacao beans. The material was mixed vigorously (30 s to 1 min) prior to addition of 300 µl extraction buffer and 60 µl 20% sodium lauroyl sarcosinate (pH 5.0). Afterwards the solution was mixed again. The mixture (~860 µl) was transferred to a 2 ml collection tube, stripped from the stainless steel beads, and incubated for 10 min at RT (without shaking). The entire volume was then added to the first RNeasy filter and the protocol was continued according to manufacturer’s instructions. In the third workflow (C), 1 ml of preheated (65°C) Sangha buffer, *i.e.* [2% (w/v) CTAB, 2% (w/v) PVP, 100 mM Tris HCl (pH 8.0), 25 mM EDTA, 2 M NaCl, 0.1% spermidine, and 2% β-mercaptoethanol], was added to 100 mg of crushed cacao beans. Following 30 min incubation at 65°C (with continuous or frequent shaking), an equal volume of chloroform:isoamylalcohol (24:1) was added and mixed for 30 s. Subsequently, the mixture was centrifuged for 20 min at 10,000 g (4°C). Next, the supernatant was transferred to a new tube and the chloroform:isoamylalcohol step was repeated. Then, the mixture was centrifuged for 10 min at 10,000 g (4°C). Finally, the supernatant was transferred to the first RNeasy filter and the protocol was continued according to manufacturer’s instructions. Comparative analysis was based on the resulting RNA yield and integrity retrieved from cryogenically crushed cacao bean samples.

### Phase 2: RNA Extraction Protocols

Five RNA extraction protocols were compared, namely (A) RNeasy^®^ Plant Mini Kit (Qiagen, Inc., Chatsworth, CA), (B) Spectrum™ Plant Total RNA Kit (Sigma Aldrich, St. Louis, MO, USA), (C) InviTrap^®^ Spin Plant Mini Kit (Stratec Molecular, Birkenfeld, Berlin), (D) Reliaprep™ RNA cell Miniprep System (Promega, Madison, WI, USA), and (E) a manual 3% CTAB-based RNA extraction protocol (Luypaert et al., 2017). Prior to each kit-based extraction, a Sangha homogenization workflow was executed as selected in phase 1. Subsequently, RNA extraction kit protocols were continued according to the manufacturer’s instructions. Comparative analysis was based on the resulting RNA yield, purity, and integrity. Extractions were executed in triplicate.

### Phase 3: RNA Preservation Conditions

In total, 13 preservation conditions differing in tissue crushing degree, preservation method, duration, and temperature were evaluated in triplicate (**Table 1**). Five of these preservation conditions were applied on both crushed and whole preserved bean samples, namely (A) 1 week at −80°C (gold standard), (B) 1 week at −20°C, (C) 3 days at 4°C, (D) 1 week of FD samples at RT, and (E) 5 weeks of FD samples at RT to assess the effect of different preservation conditions at the level of method, crushing degree, temperature, and duration. Two conditions were tested only on crushed samples: (F) 1 week at −80°C with daily shifts to −20°C and (G) 1 week at −80°C with daily shifts to 4°C to simulate temperature shifts and freeze-thaw cycles during preservation/transportation, respectively. One condition was tested on only whole bean samples: (H) 3 weeks at RT with weekly shifts to −80°C to simulate a transportation of FD samples at RT to a lab which allowed preservation at −80°C. FD was executed by freezing the material for at least 15 min at −80°C and drying them in a Alpha 1-2 LD plus freeze dryer (Christ, Osterode, Germany) (−40°C, 0.25 bar, 24 h). Comparative analysis was based on the resulting RNA yield and integrity retrieved from cryogenically crushed samples by the selected 3% CTAB-based RNA extraction protocol in phase 2.

**Table 1:** Code names and detailed description of preservation conditions tested.

T_preservation_	t_preservation_	Nomenclature 1	Tissue form	Nomenclature 2
−80°C	1 w	−80°C	C	C −80°C
			W	W −80°C
−20°C	1 w	−20°C	C	C −20°C
			W	W −20°C
4°C	3 days	4°C	C	C 4°C
			W	W 4°C
RT	1 w	FD RT 1w	C	C FD RT 1w
			W	W FD RT 1w
	5 w	FD RT 5w	C	C FD RT 5w
			W	W FD RT 5w
−80°C/ −20°C	Shifts every day for 1 week	−80°C/−20°C	C	
−80°C/4°C	Shifts every day for 1 week	−80°C/4°C	C	
RT/−80°C	Shifts every week for 3 weeks	FD RT/−80°C	W	

Nomenclature 1 is independent from tissue form. Nomenclature 2 is dependent from tissue form.

T, temperature; t, time; FD, freeze-dried; RT, room temperature; C, crushed bean; W, whole bean; w, week.

### RNA Quantity, Quality, and Integrity

Following extraction, RNA quantity was measured by the Qubit RNA HS assay kit and the Qubit^®^ 2.0 Fluorometer (Invitrogen, Carlsbad, CA). RNA quality was measured by A_260/280_ (protein contamination) and A_260/230_ (polyphenol and polysaccharide contamination) using the NanoDrop (ND) 1000 UV-Vis Spectrophotometer (NanoDrop Technologies, USA). RNA integrity was verified using the fragment analyzer™ according to the guidelines detailed in the sensitivity RNA analysis Kit, DNF-472-0500 (Advanced analytical technologies). The generated data was analyzed using PROsize 2.0^®^ (Agilent), which resulted in RNA quality numbers (RQN). The retrieved chromatograms were in addition scored visually from A to F (**Supplementary Figure S1**).

### Method Validation With RT-qPCR

Reverse transcriptase quantitative PCR (RT-qPCR) was used to validate independently the suitability of the extracted RNA from the most optimal extraction and preservation method proposed by this study. For this purpose, six cacao beans from the CCN 51 variety were sampled on-field in Ecuador and preserved at −80°C. These were freeze dried prior to express transportation (within 4 days) to Belgium and kept at −80°C until further notice.

In-house, RNA was extracted with the selected 3% CTAB extraction protocol. The obtained RNA was DNase treated using the RapidOut DNA Removal kit (ThermoFisher Scientific, Waltham, MA, USA) according to manufacturer’s protocol. The iScript™ Advanced cDNA Synthesis kit (BioRad, Richmond, CA, USA) was used to synthesize cDNA by means of 500 ng RNA input.

Five cacao specific reference genes (**Supplementary Table S1**) (Pinheiro et al., 2011; Liu et al., 2013) were analyzed in duplicate. For qPCR, a Tecan Freedom EVO^®^ robot was used to distribute a 1:3 cDNA : SsoAdvanced™ Universal SYBR^®^ Green Supermix (BioRad, Richmond, CA, USA) mixture and primermixes (1.25 µM) in each well of a Hard-Shell^®^ 384-well PCR plate (BioRad, Richmond, CA, USA). Amplification was performed by a LightCycler^®^ 480 System (Roche, Basel, Switzerland) by means of the following thermal cycling conditions: incubation at 95°C for 2 min, 40 cycles of 95°C for 5 s; 60°C for 30 s, and 72°C for 1 s. Melting curves for each amplicon were determined between 65 and 95°C, followed by a cooling step to 4°C. Experiments included a negative control (no template), a no reverse-transcriptase (noRTs) control, and a positive control (1 ng cacao leaf DNA and DNA specific primers in a similar setup).

### Statistical Analysis

The mean RNA quantities and integrities resulting from different extraction conditions were compared between and within groups by Welch corrected t-tests, one-way and two-way ANOVA test using Tukey *post hoc* procedures. Normality and homoscedasticity assumptions were verified visually by means of diagnostic plots of the residuals. In case assumption of homoscedasticity was violated, a Welch corrected one-way ANOVA test was performed with Dunett’s T3 *post hoc* tests. The significance level was set at 0.05. Statistical analysis was performed using IBM-SPSS version 22.

## Results

### Impact of Sample Homogenization on RNA Extraction Efficiency

In the first phase (**Figure 1**), three sample homogenization procedures were compared. The commercially available RNeasy^®^ plant mini kit (Qiagen) was used for extraction. Pulverization was increased using stainless steel beads. Three chemical homogenization buffers were tested, namely: (A) RLT buffer (Qiagen), (B) guanidine thiocyanate-based MacKenzie buffer (MacKenzie et al., 1997), and (C) CTAB-based Sangha buffer (Sangha et al., 2010). No significant differences could be observed at the level of RNA quantity and quality between the MacKenzie and Sangha protocols (**Supplementary Table S2**), though the Sangha buffer consistently generated higher yields. Remarkably, the unmodified protocol using the RLT buffer resulted in insufficient RNA yields and was therefore excluded from further statistical analysis. The RNA quantity ranged from 6.43 (MacKenzie) to 15.5 ng/µl (Sangha). A_260/280_-based quality ratios ranged from 1.48 (MacKenzie) to 2.08 (Sangha), while A_260/230_ values ranged from 1.14 (Sangha) to 1.89 (MacKenzie). Overall, both buffers resulted in acceptable purity extracts. However, the Sangha buffer showed higher protein removing reproducibility (A_260/280_), whereas MacKenzie was more consistent in removing polysaccharide contaminants (A_260/230_). Overall, the RNA extraction efficiency of the RNeasy^®^ Plant Mini Kit was most improved when using the CTAB-based Sangha buffer.

### Comparison of RNA Extraction Protocols

**Figure 2:**
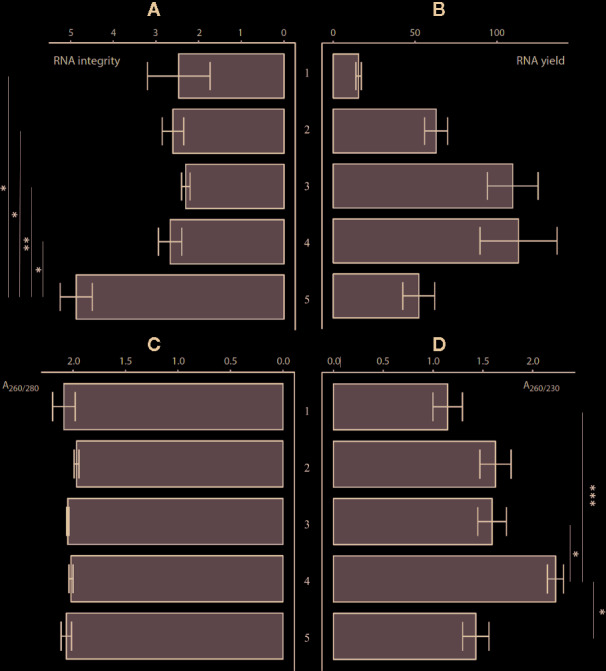
The comparison of RNA extraction protocols (n = 3) at the level of: **(A)** RNA integrity, **(B)** RNA yield (ng/µl), **(C)** A_260/280_ purity, and **(D)** A_260/230_ purity [Error bars indicate mean (µ) ± standard error of mean (SEM)]. (1) RNeasy^®^ Plant Mini Kit ac. Sangha, (2) Spectrum™ Plant Total RNA Kit ac. Sangha, (3) InviTrap^®^ Spin Plant Mini Ki ac. Sangha, (4) Reliaprep™ RNA cell Miniprep System ac. Sangha, and (5) 3% CTAB protocol (Luypaert et al., 2017). *p < 0.05, **p <0.01, ***p < 0.001. More details on significance can be found in **Supplementary Table S4**.

In the second phase (**Figure 1**), we compared four commercially available kits (the RNeasy Plant Mini Kit, Spectrum Plant Total RNA Kit, InviTrap Spin Plant Mini Kit, and the Reliaprep RNA cell Miniprep System) combined with the Sangha homogenization workflow described above. A manual 3% CTAB-based extraction protocol was assessed as well (Luypaert et al., 2017). All protocols were evaluated at the level of yield, quality, and integrity. A one-way analysis of variance (ANOVA) demonstrated significant differences between the methods in the context of RNA quantity, quality, and integrity (**Supplementary Tables S3** and **S4**, **Figure 2**). Measured RNA yields ranged on average between 15.5 (RNeasy) and 113.13 ng/µl (Reliaprep). Although an overall significant effect of the extraction protocol has been detected (p = 0.007), *post hoc* testing could not reveal which protocol was causing this. Polyphenol and polysaccharide removal efficiencies (A_260/230_) ranged between 1.15 (RNeasy) and 2.23 (Reliaprep), and showed great consistency within each protocol. The Reliaprep resulted in significant higher polysaccharide removal efficiencies in contrast to the other kits (p < 0.05), with exception of the Spectrum protocol (p = 0.061). Protein removal efficiencies (A_260/280_), on the other hand, were very similar for all protocols with values ranging from 1.96 (Spectrum) to 2.10 (3% CTAB), reaching optimal removal efficiencies (A_260/280_ = 2). The measured RNA integrity was overall low ranging from 2.3 (InviTrap) to 4.2 (3% CTAB), with the 3% CTAB protocol showing significant higher integrity (p < 0.03) relative to all other workflows (**Supplementary Table S4**). Overall, the 3% CTAB protocol (Luypaert et al., 2017) resulted in RNA with significantly higher integrities. In addition, the method is more straight-forward and more cost-efficient compared to the other commercial kits tested.

### Effect of Tissue Form, Preservation Method, and Temperature

**Figure 3:**
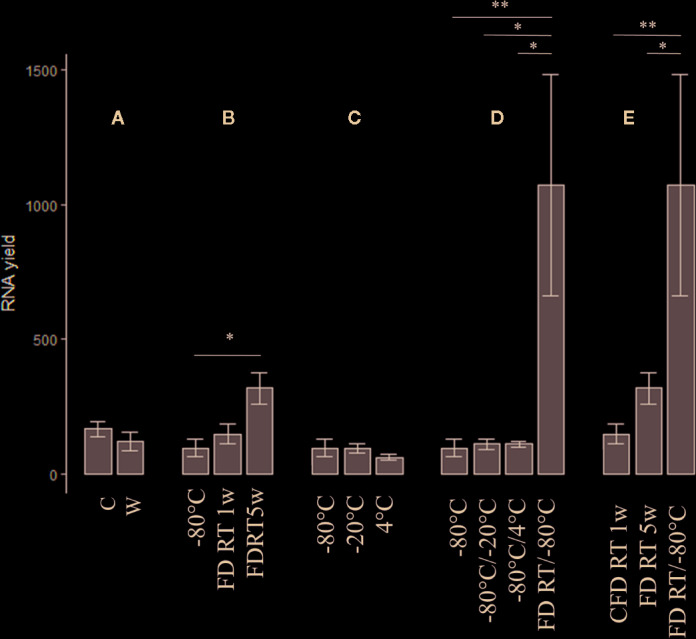
The impact of tissue form (crushed *vs* whole) (n = 15), FD treatment, temperature, and temperature shifts assessed on extraction yield (n = 3). A = C *vs.* W, B = FD conditions *vs.* gold standard (−80°C), C = suboptimal temperature conditions (−20°C, 4°C) *vs.* gold standard, C = Temperature shifts *vs.* gold standard, D = Changing preservation conditions vs. gold standard and E = Comparison of all FD conditions.. Nomenclature is as described in **Table 1**. C, crushed bean; W, whole bean; FD, freeze-dried; RT, room temperature; w, week(s). (Error bars indicate µ ± SEM) *p < 0.05, **p < 0.01. Details of significance levels between conditions can be found in **Supplementary Tables S5** and **S6A**.

**Figure 4:**
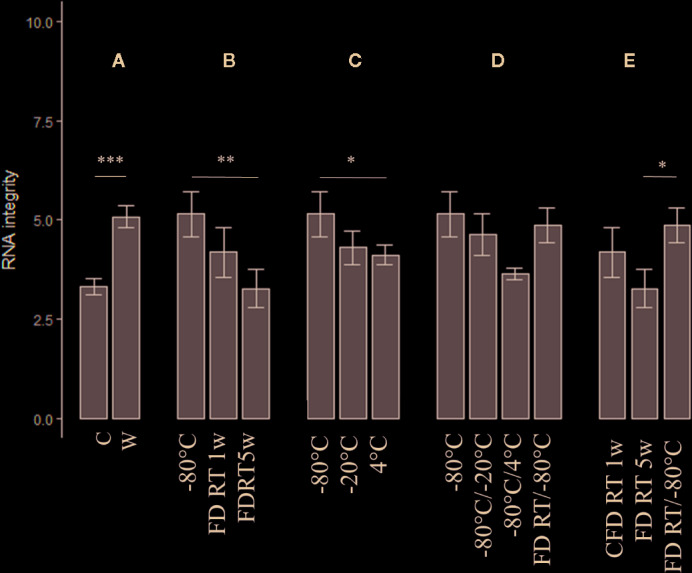
The impact of tissue form (crushed *vs* whole) (n = 15), FD treatment, temperature, and temperature shifts assessed on RNA integrity (n = 3). A = C *vs.* W, B = FD conditions *vs.* gold standard (−80°C), C = suboptimal temperature conditions (−20°C, 4°C) *vs.* gold standard, C = Temperature shifts *vs.* gold standard, D = Changing preservation conditions vs. gold standard and E = Comparison of all FD conditions.. Nomenclature is as described in **Table 1**. C, crushed bean; W, whole bean; FD, freeze-dried; RT, room temperature; w, week(s). (Error bars indicate mean ± standard error) *p < 0.05, **p < 0.01. Details of significance levels between conditions can be found in **Supplementary Tables S5** and **S6**.

Since on-site wet-lab analyses are (mostly) unfeasible in cacao-producing countries, we aimed to optimize preservation conditions compatible with prolonged shipping procedures. In phase 3 (**Figure 1**), the impact of tissue form (crushed *vs* whole), FD treatment, temperature, and temperature shifts was assessed on RNA integrity and extraction yield. All raw data and statistical studies are documented in **Supplementary Tables S5** and **S6**, respectively. An overview of extraction yield and integrity is depictured in **Figures 3** and **4**, respectively. The code names (abbreviations) of each tested preservation condition are listed in methodology (**Table 1**).

For evaluation, the 3% CTAB protocol was used due to its higher integrity values upon extraction. Here, we mainly focused on RNA yield and integrity, as purity seemed to be less influenced by preservation conditions and rather extraction method-dependent (as indicated by the greater purity consistency stated within an extraction method). Besides, the RNA, together with its impurities, is typically diluted to a workable concentration prior to downstream processing. Moreover, the UV spectroscopy based purity measurements lack accuracy and their relevance should be considered carefully. In general, A_260/280_ and A_260/230_ values ranged from 1.91 to 2.15, and 0.96 to 2.11, respectively (**Supplementary Table S5**). For a more balanced assessment of RNA integrity, evaluations were based on a combined analysis of RQN values and visual scoring (from A to F) of electropherogram peak shapes generated by the fragment analyzer™ (Advanced analytical technologies, **Supplementary Figure S1**).

The effect of tissue form—crushed *versus* whole beans—on RNA quantity and integrity was assessed independently from temperature as depictured in **Figures 3** and **4B**, respectively. In general, crushed samples resulted in consistently higher RNA yields relative to whole beans, although no statistical difference could be observed (p = 0.557). Only for the non-FD −20°C condition, the crushed sample showed significant higher yields in comparison to the whole bean (p = 0.006, column “C *vs* W” in **Supplementary Table S5**). RNA integrity, on the other hand, was significantly higher for the whole preserved bean samples in comparison to the corresponding crushed samples (p < 0.001). This was especially the case for FD samples stored at RT during five weeks (FD RT 5w; p = 0.015) and non-FD samples continuously stored at 4°C (p = 0.040, column “C *vs* W” in **Supplementary Table S5**).

Since FD transport of samples at RT is generally considered as a good alternative for the more complex and costly shipments at −80°C, we assessed the impact of FD samples stored for 1 (FD RT 1w) and 5 weeks (FD RT 5w) at RT on RNA extraction characteristics (independently from tissue form) (**Supplementary Tables S5** and **S6A**, **Figures 3** and **4B**). In general, prolonged preservation of FD samples at RT resulted in higher extraction yields (96.57 ng/µl for FD 1w *versus* 342.07 ng/µl for FD 5w, **Supplementary Table S5**). For both FD conditions higher RNA quantities could be observed in comparison to the −80°C reference sample. However, only between the FD 1w and FD 5w samples significant differences could be observed (p = 0.05, **Supplementary Table S6A**, **Figure 3**). Longer preservation of FD samples at RT was correlated with decreasing integrity values. RNA integrity for both FD conditions was generally lower than preservation at −80°C (RQN = 6.07), and differed significantly for the FD 5w condition (p = 0.005, **Figure 4B**, **Supplementary Table S6A**).

The impact of preservation temperatures, −80, −20, and 4°C, was assessed independently from tissue form (**Figures 3** and **4C**). RNA quantities ranged from 50.3 (4°C) to 130 ng/µl (−20°C) (**Supplementary Table S5**), but no significant differences could be observed (overall p = 0.569, **Supplementary Table S6B**). RNA integrity values (overall p = 0.034, **Supplementary Table S6B**) increased with decreasing preservation temperature (µ_4_ = 4.12, µ_-20_ = 4.3, and µ_-80_ = 5.15, **Supplementary Table S5**), differing significantly between samples stored at −80 and 4°C (p = 0.030, **Supplementary Table S6**).

Temperature changes between these conditions did not seem to have a significant effect on RNA integrity (overall p = 0.234, **Figure 4D**, **Supplementary Table S6C**), although temporary temperature shifts to 4°C generally resulted in lower integrity values (**Supplementary Table S5**). The RNA yields did not differ significantly between then non-FD conditions (**Figure 3**, **Supplementary Table S6C**).

In contrast, the FD condition with temperature shifts between RT and −80°C resulted in significantly higher RNA quantities (p ≤ 0.019) (**Figure 3**, **Supplementary Table S6D**). When comparing the latter condition with the other FD conditions, significantly higher yields were obtained relative to FD RT 1w and FD RT 5w (p = 0.008 and p = 0.025, respectively). The RNA integrity for the FD samples undergoing weekly RT/−80°C temperature shifts was significantly increased in comparison to the FD RT 5w sample (p = 0.033, **Figure 4E**, **Supplementary Table S6D**).

### Compatibility of Extracted RNA With Downstream Processing

Compatibility of the 3% CTAB-based DNase treated RNA extracts on six independent FD RT transported CCN cacao bean samples was assessed using RT-qPCR. All six samples were amplifiable for every reference gene assessed with average Cq values ranging from 26.45 ± 1.07 (*GADPH*) to 28.62 ± 0.85 (*ACT-L*) (**Supplementary Table S7**). This indicates the applicability of the RNA for downstream processing. In addition, the RNA has been used successfully in a sequencing set-up using the TrueSeq RNA sample preparation kit (Illumina, San Diego, California, USA) (data not shown).

## Discussion

Previous studies compared different RNA extraction protocols on plant tissues from various types and species. However, to the best of our knowledge, no comparative evaluation of RNA integrity, quality, and yield upon extraction from recalcitrant cacao bean tissue has been performed. Besides, no optimal RNA-stable preservation and transportation method for cacao beans has been described. We will discuss the impact of (1) homogenization, (2) extraction, and (3) preservation on the retrieved cacao bean RNA with insights of interest to every plant geneticist. Finally (4), we will select the most optimal cacao bean RNA extraction and preservation workflow suitable for overseas analyses.

### The Impact of Sample Homogenization on RNA Extraction Efficiency

All RNA extraction protocols start by homogenizing the sample of interest. This entails mechanical (pulverization) and chemical disruption (lysis using extraction buffers) of the tissue. Pulverization by means of a sonicator, a mixer mill, glass and stainless steel beads, or a mortar and pestle, divides the sample into a multitude of smaller chunks, thereby increasing the contact surface of the lysis buffer resulting in higher RNA yields. This is especially important for heavily encased seeds and tough tissues (Varma et al., 2007; Burden, 2008).

In this study, cryogenic grinding by means of mortar and pestle was performed, followed by a stainless steel bead based homogenization. For chemical disruption three different chemical disruption (homogenization) buffers were compared in the context of RNA yield and quality upon the RNeasy^®^ plant mini kit based extraction (Phase 1). This kit is commonly used for plant samples, including cacao leaves (Verica et al., 2004), and has shown to result in high yielding and high quality RNA extraction of difficult tissues (*e.g.* seed and fruit tissue) (Sangha et al., 2010; Kalinowska et al., 2012).

We demonstrated that both the guanidine thiocyanate-based MacKenzie and the CTAB-based Sangha buffer increased cacao bean RNA yields. Higher yields were observed for the Sangha buffer in comparison with the MacKenzie buffer, which is consistent with previous research (Sangha et al., 2010). The unmodified RNeasy^®^ protocol, making use of the RLT buffer, seemed incompatible with the complex recalcitrant cacao bean tissue, since it failed to generate measurable RNA quantities. Based on these results and observation in citrus fruits, we hypothesize that guanidine thiocyanate, present in the RLT and MacKenzie buffers, is less efficient in lysing cacao bean cells and is probably unable to dissociate RNA from RNA-polysaccharide complexes (Tao et al., 2004). In contrast, PVP and CTAB-based Sangha buffers seem to be more compatible with cacao bean tissue and resulted in higher extraction yields, especially when preheated as was the case in this study (Vasanthaiah et al., 2008). Similar conclusions on CTAB-based buffers have been drawn by previous studies on recalcitrant and/or highly contaminated tree plant tissues (Vasanthaiah et al., 2008; Sangha et al., 2010; Ling et al., 2013; Zhu et al., 2013; Luypaert et al., 2017). RNA yields could further be increased by optimizing sample input, through secondary elutions or by using PVP during grinding (Gesteira et al., 2003; Rodrigues et al., 2007; Vasanthaiah et al., 2008).

Also differences in RNA purities were observed between the Sangha and the MacKenzie buffer. PVP, combined with NaCl and CTAB (Sangha) or guanidium thiocyanate (MacKenzie), prevents polyphenol oxidization and aids in contaminant-removal from nucleic acid extracts (Mornkham et al., 2013; George, 2018). Although PVP is present in both the Sangha and the MacKenzie buffer, the A_260/230_ ratios were lower for the Sangha buffer, indicating polysaccharide and polyphenol contamination. There is, however, no consensus on the acceptable lower limit of this ratio, nor has it been demonstrated to significantly affect the reliability of downstream applications (De Keyser et al., 2013). If needed, purity may be further increased by additional extraction buffer washing steps or extra ethanol precipitations during the purification. A_260/280_ ratios—indicative for protein removal—were sufficiently high (>1.4) for all protocols, although scores were markedly higher for the Sangha buffer. The low protein contamination is mainly due to the efficient protein degrading properties of β-mercaptoethanol, the complexation by CTAB or guanidium isothiocyanate, and the RNA specificity of the extraction protocol. Protein removal may be further increased by using preheated buffers and additional phenol and chloroform purification steps. However, the latter has proven to be more toxic, damage poly (A)+ RNA and generate lower yields (Chang et al., 1993; Varma et al., 2007; Yockteng et al., 2013). Although defatting cacao beans prior to RNA extraction could also further increase purity, it may affect RNA integrity (Varma et al., 2007; Ramos et al., 2014). Another important factor is the buffer volume added during homogenization according to the recommendation of the homogenization protocols (Sangha and MacKenzie). According to MacKenzie only 300 µl of its buffer should be added, while Sangha recommended to use 1 ml of its buffer, possibly affecting dilution and extraction efficiency.

Altogether, these results suggest that the type of chemical disruption and the level of pulverization during homogenization impacts bean RNA extraction efficiencies. For cacao beans, we recommend Sangha-based homogenization with pulverization, but other methods might prove more efficient for other tissue types or plants.

### The Impact of the RNA Extraction Protocol on RNA Quality

Next, we compared four commercially available RNA extraction kits and a manual 3% CTAB protocol, supplemented with the Sangha-based homogenization workflow (phase 2). Although kits are time-efficient, they are not always designed for polyphenol- and polysaccharide-rich plant tissues. Such contaminants may result in viscous extracts blocking the silica columns drastically influencing extraction efficiencies (Rodrigues et al., 2007; Varma et al., 2007; Sangha et al., 2010). The tested protocols did not generate significantly different RNA quantities. However, differences at the level of purity and integrity could be observed. Since similar disruption and extraction buffers were used in all protocols, we suspect the differences can be attributed to differences in RNA isolation and purification.

The commercial kits use silica gel-based filter columns to separate the debris from the nucleic acids, while the 3% CTAB protocol uses centrifugation and selective precipitation by LiCL. Overall, almost no protein contamination was detected for any of the methods. In contrast, A_260/230_ purities seemed compromised (<2.00), except for the Reliaprep protocol. This suggests that A_260/230_ purities are not influenced by how RNA is separated from additional contaminants (silica gel column filters or pelletation), but how it is homogenized. The higher purity levels observed for the Reliaprep protocol could be attributed to the chloroform:isoamylalcohol based purification used in this kit, instead of the pure-chloroform workflow applied in the others. Furthermore, the Reliaprep protocol uses an additional isopropanol RNA precipitation step before RNA capture on columns. Similar 3% CTAB based approaches have already been applied successfully on cacao tissues (Argout et al., 2008). Further increase of purities might be obtained by using poly-vinyl-poly pyrrolidone (PVPP) or polyethylene glycol (PEG) instead of PVP, by additional filtration, chloroform purification, or ethanol precipitation steps, or resuspension in ice-cold isopropanol or 3M sodium acetate (Argout et al., 2008; Sangha et al., 2010; Gudenschwager et al., 2012; Luypaert et al., 2017). Nevertheless, extracts from all RNA isolation kits tested seem to be compatible with downstream analyses such as cDNA library construction, RT-qPCR and sequencing (data not shown) (Jones et al., 2002; Verica et al., 2004; Gesteira et al., 2007).

RNA integrity is considered as the most important RNA extraction efficiency parameter, since low integrity can dramatically impact downstream results (Fleige and Pfaffl, 2006; Pinheiro et al., 2011; Die and Román, 2012; Johnson et al., 2012). However, this is often neglected in biological studies, violating the MIQE-guidelines (Bustin et al., 2009). RNA integrity is most accurately determined by capillary electrophoresis, which allows for visual interpretation and reports RQN- or RIN-values. In general, values greater than eight are commonly associated with good integrity. However, for plant samples aberrant recommendations can be found in literature. Pereira et al. (2017) considered RIN ≥6.5 adequate for RT-qPCR based transcriptomic studies, while Weißbecker et al. (2017) and Fleige and Pfaffl (2006) recommended RIN values greater than 5 and 4.5, respectively (Fleige and Pfaffl, 2006; Pereira et al., 2017; Weißbecker et al., 2017). Furthermore, several studies have demonstrated an overall underestimation of RNA degradation and integrity when relying solely on RIN quality values in plants (Johnson et al., 2012; De Keyser et al., 2013; García-baldenegro et al., 2015). To compensate for this, we adopted a visual scoring (from A to F) of the electropherograms generated by the fragment Analyzer™ (Advanced analytical technologies) in addition to RQN scores. The latter is in concordance with the opinion of Die and Román (2012). The highest RQN values and clearest plots were observed for the 3% CTAB protocol, all other protocols showed low or sometimes unmeasurable integrity scores. The rather low measured integrity values could be attributed to the prolonged and uncontrolled in-pod transportation of the beans. Nevertheless, even samples having RQN lower than four could be successfully processed downstream (data not shown). Although the Reliaprep protocol generated the highest RNA yields and purities on cacao bean tissue, we opted for the 3% CTAB workflow due to its significantly higher RNA integrity levels.

### The Importance of Preservation and Transportation

RNA stability highly depends on the type of preservation in addition to sample collection, harvesting, handling, transportation, and the used extraction protocol. The gold standard for RNA preservation is immediate liquid nitrogen based cryopreservation (−80°C) after flash freezing (Auer et al., 2014). However, this is most of the time unfeasible on-site at farms in the cacao-producing countries. Since higher-temperature shipping (−20°C or above) of whole-tissue samples could result in a reduction of RNA quality and integrity, as ribonucleases activity has still been observed at −20°C and even at −70°C (Fabre et al., 2014). Therefore alternative shipping and preservation workflows needed to be considered (phase 3). Shipping at −20°C is feasible and seems to result in adequate qualities and integrities. However, has the potential to result in lower RNA integrities if freeze-thaw cycles occur (4 or −80°C/4°C) (Hernandez et al., 2009; García-baldenegro et al., 2015), when suffering from shipment delays or suboptimal packaging (Varma et al., 2007; Hernandez et al., 2009).

As alternative, we assessed the compatibility of FD with overseas shipment. FD is used for a wide range of applications within the food industry, pharmacy, and biotechnology. It has been used successfully for RNA preservation in grapevine buds (García-baldenegro et al., 2015), banana tissue (Lassois et al., 2009), and tea leaves (Jaiprakash et al., 2003). However, complete RNA degradation has been observed in FD cotton, indicating the tissue- and species-specificity of the technique (Pearson et al., 2006). In comparison to the other preservation conditions, significantly higher yields could be obtained through FD. RNA integrity levels were relatively low for FD samples kept at RT. This was unexpected since the decreased water content should inactivate proteolytic enzymes and nucleases, thereby reducing cellular component degradation (Santos et al., 2014; García-baldenegro et al., 2015). We assume that this could originate from suboptimal drying and inadequate closing of the sample vials, resulting in the uptake of water from the humid air during transport (García-baldenegro et al., 2015). Furthermore, integrity of FD samples showed inversely proportional with duration of the preservation at RT.

Tissue form, *i.e.* whole or crushed, will most likely also have an impact during transport or preservation (Johnson et al., 2012; George, 2018). We observed that (both FD and non-FD) crushed samples resulted in higher RNA yields in comparison to whole bean preservation. This could be attributed to increased cell lysis during the crushing and preservation of pulverized samples, whereas whole beans were better protected. In contrast, the integrity was consistently higher for whole bean preserved samples. This is probably due to the release of RNases during crushing and the increased surface area exposed, prior to preservation. For whole bean samples, extraction buffer is added immediately after crushing (homogenization), allowing the chemical agents to quickly inhibit ribonuclease activity (Claros and Cánovas, 1999).

### The Most Suitable RNA Preservation and Extraction Protocol for Gene Expression Analysis

When considering all results, and taking into account that RNA integrity potentially has the largest impact on downstream processing, we believe that FD whole beans (with in-house preservation at −80°C upon arrival) combined with 3% CTAB extraction protocol is the optimal setup to obtain high-quality RNA extracts from cacao beans sampled in countries with limited resources. This procedure is furthermore perfectly compatible with overseas shipping and gene expression analysis. With these findings the complexity of performing genetic studies on cacao beans from foreign countries is reduced. The findings will contribute to cacao bean transcriptome-based studies conducted worldwide, as long as dry ice (−80°C) and a freeze dryer is available and the transportation at RT within a week to a resource-full lab is guaranteed. Moreover, this study provides insights on the importance of RNA extraction and preservation conditions on RNA integrity relevant to the entire plant science society, such as crushing degree, homogenization, extraction method, freeze-drying, preservation time, freezing conditions, and freeze-thaw cycles. Overall, an outline for studies, experiencing troubles with RNA extraction, preservation, and/or transportation, is provided.

## Data Availability Statement

The datasets used and/or analyzed during the current study are available in supplementary or on reasonable request from the corresponding author.

## Author Contributions

JW wrote the manuscript. JW and SL conceived and designed the study as written in the main manuscript text. JW and DT equally contributed to the practical work conducted for this study. Both HE and HR offered their plant-associated expertise needed for this study, while SL advised on the genetic part. JV assisted on the statistical aspect of the paper. KM and KD supported and guided this study with their essential expertise, network, and resources in the field of cacao. All authors contributed to the article and approved the submitted version.

## Funding

This work was funded by special research fund (BOF) scholarship [BOF16/DOC/338 (JW)] from Ghent University. Mobility to Ecuador and Ghana was funded by the Commission scientific research (CWO) (BOFFF22014000501) at the Faculty of Bioscience Engineering and the Research Foundation Flanders (FWO) (V405218N), respectively.

## Conflict of Interest

The authors declare that the research was conducted in the absence of any commercial or financial relationships that could be construed as a potential conflict of interest.
