# Supplementary material for: A Combined RNA Preservation and Extraction Protocol for Gene Expression Studies in Cacao Beans
Source: Front Plant Sci. 2020 Jun 30;11:992. doi: 10.3389/fpls.2020.00992 (PMC7338848; doi:10.3389/fpls.2020.00992)

Supplementary Material

1 Supplementary Tables

Supplementary Table S1. Cacao specific reference genes and primer sequences, used to test amplifiability of RNA samples in RT-qPCR set-up.

Full name	Gene	Primer	Source
Actine	*ACT_P*	F: TCCTCTTCCAGCCATCTCTC<br>R:TCTCCTTGCTCATTCGGTCT	Pinheiro et al., 2011
	ACT_L	F:AGCTGAGAGATTCCGTTGTCCAGA<br>R:CCCACATCAACCAGACTTTGAGTTC	Liu et al., 2013
Malate dehydrogenase	*MDH*	F: AAAATGGAGTTGGTGGATGC<br>R: AACCATGACTGCGATGTTGA	Pinheiro et al., 2011
Glyceraldehyde 3-phosphate dehydrogenase	*GAPDH*	F: GATGCTCCTATGTTTGTTGTGG<br>R: TCTTCCTCCTCTCCAGTCCTT	
Ribosomal protein L35	*RpL35*	F: CCTAACAAGCTTACCAAGATAAAG<br>R: TTATTCTTATATGCCTCCCTCAGTG	

Supplementary Table S2. Statistical evaluation on the impact of homogenization on retrieved RNA quality and quantity from cacao bean tissue using the RNeasy® Plant Mini Kit (n = 3). Results figured as mean (µ) and standard error of mean (SEM).

Extraction protocol	Homogenization	Quantity		Quality			
		yield (ng/µL)	*p value*	$A_{260/280}$	*p value*	$A_{260/230}$	*p value*
**RNeasy® Plant Mini Kit**	*RLT*	< 0.02 *		1.65 ± 0.08		1.31 ± 0.41	
	MacKenzie et al. (1997)	6.43 ± 3.25	0.096	1.48 ± 0.27	0.108	1.89 ± 0.46	0.423
	Sangha et al (2010)	15.50 ± 1.66		2.08 ± 0.11		1.14 ± 0.15	

* below detection limit, protocols are compared using two sample t-tests (p value: two-tailed).

Supplementary Table S3. Statistical evaluation on the impact of RNA extraction method on retrieved RNA quality, quantity and integrity from cacao bean tissues (n = 3). Post hoc tests are figured in Appendix 3. Figured as µ and SEM. Light grey p values: $p < 0.05$

**Cacao bean RNA extraction protocol**	**Quantity**		**Quality****				**Integrity**	
	yield (ng/µL)	*p value*$^{\$}$	$A_{260/280}$	*p value*	$A_{260/230}$	*p value*	*RQN****	*p value*
RNeasy® Plant Mini Kit ac. Sangha	15.5 ± 1.66	0.007	2.08 ± 0.11	0.608	1.15 ± 0.15	0.003	2.46 ± 0.73	0.006
Spectrum™ Plant Total RNA Kit ac. Sangha	62.87 ± 7.00		1.96 ± 0.02		1.63 ± 0.20		2.6 ± 0.25	
InviTrap® Spin Plant Mini Kit ac. Sangha	109.63 ± 15.52		2.05 ± 0.01		1.59 ± 0.15		2.3 ± 0.10*	
Reliaprep™ RNA cell Miniprep System ac. Sangha	113.13 ± 23.52		2.02 ± 0.02		2.23 ± 0.08		2.67 ± 0.27	
3% CTAB protocol (19)	52.23 ± 9.74		2.10 ± 0.05		1.43 ± 0.13		4.2 ± 0.38	

* calculations are based on two biological replicates instead of three, ** absorbance ratios around 2.0 indicate pure samples, *** values higher than 4.0 were considered suitable for preliminary research, $^{\$}$ Reported p values are Welch corrected

Supplementary Table S4. Comparing different extraction methods at the level of quality, quantity and integrity (n = 3) using one-way ANOVA, whether or not, with Welch correction (in case of violation of homoscedasticity) and standard or Dunett's T3 post hoc procedures, respectively. Light grey p values: $p < 0.05$

**Comparison of RNA extraction protocols**					
Quantity[$]					
**Extraction method**	*RNeasy®*	*Spectrum™*	*InviTrap®*	*Reliaprep™*	*3% CTAB*
RNeasy®		0.069	0.094	0.191	0.221
Spectrum™			0.319	0.536	0.969
InviTrap®				1.00	0.214
Reliaprep™					0.414
3% CTAB					
Quality					
$A_{260/280}$					
**Extraction method**	*RNeasy®*	*Spectrum™*	*InviTrap®*	*Reliaprep™*	*3% CTAB*
RNeasy®		0.554	0.988	0.932	0.988
Spectrum™			0.813	0.932	0.724
InviTrap®				0.998	1.00
Reliaprep™					0.988
3% CTAB					
$A_{260/230}$					
**Extraction method**	*RNeasy®*[A]	*Spectrum™*[A,B]	*InviTrap®*[A]	*Reliaprep™*[A,B]	*3% CTAB*[A]
RNeasy®		0.163	0.209	0.001	0.593
Spectrum™			1.000	0.061	0.836
InviTrap®				0.047	0.906
Reliaprep™					0.012
3% CTAB					
Integrity					
**Extraction method**	*RNeasy®*[A]	*Spectrum™*[A]	*InviTrap®*[A]	*Reliaprep™*[A]	*3% CTAB*[B]
RNeasy®		0.999	0.998	0.996	0.013
Spectrum™			0.983	1.000	0.018
InviTrap®				0.965	0.008
Reliaprep™					0.021
3% CTAB					

[$] due to a potential violation of the assumption of homoscedasticity the Welch corrected p value is reported and Dunett's T2 post hoc tests were conducted. Homogeneous subsets are divided in A or B.

Supplementary Table S5: Statistical comparison of different preservation conditions in extracting qualitative and quantitative RNA of good integrity from cacao bean tissue (n=3). Two paired comparisons specific within and between methods can be found in Supplementary Table S6. More information on temperature conditions in Figure 3. Visual scoring (A to F) is based on the electropherogram peak shapes generated by the fragment analyzer™ (Advanced analytical technologies) as explained in more detail in Supplementary Figure S1. Light grey p values: $p < 0.05$

**Temperature conditions**[!]	**Quantity**		**Quality***		**Integrity**		
	yield (ng/µL)	*p value* C vs W	$A_{260/280}$	$A_{260/230}$	*RQN***	*p value* C vs W	*Visual scoring* [$]
C -80°C	136.6 ± 61.73	0.312	2.09 ± 0.04	1.92 ± 0.15	4.87 ± 0.63	0.314	B,B,A
W -80°C	53.8 ± 3.99		1.9 ± 0.09	1.00 ± 0.07	6.07 ± 0.82		B,A,B
C -20°C	130 ± 10.00	0.006	2.06 ± 0.02	1.43 ± 0.13	3.5 ± 0.15	0.062	A,B,B
W -20°C	58.3 ± 6.40		1.99 ± 0.04	1.09 ± 0.13	5.1 ± 0.46		B,B,B
C 4°C	72.53 ± 18.98	0.387	2.02 ± 0.01	1.74 ± 0.06	3.63 ± 0.18	0.040	B,C,C
W 4°C	50.3 ± 11.95		1.95 ± 0.063	0.96 ± 0.05	4.6 ± 0.25		B,C,B
C FD RT 1w	198.57 ± 62.51	0.239	1.99 ± 0.05	1.45 ± 0.23	3.03 ± 0.35	0.070	E,C,C
W FD RT 1w	96.57 ± 23.73		1.91 ± 0.03	0.96 ± 0.5	5.33 ± 0.73		B,B,B
C FD RT 5w	293.33 ± 52.07	0.728	2.15 ± 0.01	2.11 ± 0.03	2.23 ± 0.09	0.015	B,D,D
W FD RT 5w	342.07± 115.61		2.08 ± 0.02	1.63 ± 0.12	4.3 ± 0.31		C,C,D
W RT/-80°C	1073.33 ± 410.58		2.09 ± 0.04	1.62 ± 0.16	4.87 ± 0.44		B,C,C
C -80°C/-20°C	110.37 ± 19.82		2.09 ± 0.02	1.66 ± 0.08	4.63 ± 0.53		A,B,B
C -80°C/4°C	110.23 ± 11.35		1.91 ± 0.09	1.35 ± 0.10	3.63 ± 0.15		C,B,C

C= crushed bean, W = whole bean, FD = freeze-dried, RT = room temperature. P values were calculated, between crushed and whole (C vs W) bean tissue sample within one temperature condition using Welch corrected T-tests,. [$] Visual score system representing all triplicates, shown in Supplementary figure S1. * Absorbance ratios around 2 indicate pure samples, ** Values higher than 4.0 were considered adequate. [!]

Supplementary Table S6. Comparing different preservation conditions at the level of quantity and integrity (n = 3) using two-way ANOVA and standard post hoc procedures, independent from tissue form. More information can be found in Supplementary Table S4. Light grey p values: $p < 0.05$

**A) Temperature vs Freeze-dried conditions (T & FD)**					
Quantity (p = 0.011)					
**Preservation method**	*-80°C [A]*	*FD RT 1w [A]*	*FD RT 5w [B]*		
-80°C		0.697	0.11		
FD RT 1w			0.05		
FD RT 5w					
Integrity (p = 0.006)					
**Preservation method**	-80°C [B]	*FD RT 1w [A]*	*FD RT 5w [B]*		
-80°C		0.091	0.005		
FD RT 1w			0.260		
FD RT 5w					
**B) T conditions (T)**					
Quantity (p = 0.599)					
**Preservation method**	*-80°C*	*-20°C*	*4°C*		
-80°C		0.999	0.625		
-20°C			0.646		
4°C					
Integrity[3] (p = 0.034)					
**Preservation method**	*-80°C [B]*	*-20°C [A,B]*	*4°C [A]*		
-80°C		0.088	0.030		
-20°C			0.749		
4°C					

T= Temperature, t = time, RT = Room Temperature. Homogeneous subsets are divided in A or B.

Supplementary Table S6. continued

**C) Temperature changes**					
Quantity (p = 0.022)					
**Preservation method**	-80°C[A]	*-80°C/-20°C[A]*	*-80°C/4°C[A]*	*FD RT/-80°C[B]*	
-80°C		1.000	1.000	0.007	
-80°C/-20°C			1.000	0.019	
80°C/4°C				0.019	
FD RT/-80°C					
Integrity (p = 0.234)					
**Preservation method**	*-80°C*	*-80°C/-20°C*	*-80°C/4°C*	*FD RT/-80°C*	
-80°C		0.631	0.092	0.817	
-80°C/-20°C			0.604	0.991	
-80°C/4°C				0.442	
FD RT/-80°C					
**D) Freeze drying (FD)**					
Quantity (p = 0.014)					
**Preservation method**	*FD RT 1w[A]*	*FD RT 5w[A]*	*FD RT/-80°C[B]*		
FD RT 1w		0.667	0.008		
FD RT 5w			0.025		
FD RT/-80°C					
Integrity (p= 0.160)					
**Preservation method**	*FD RT 1w[A]*	*FD RT 5w[A]*	*FD RT/-80°C[B]*		
FD RT 1w		0.139	0.437		
FD RT 5w			0.033		
FD RT/-80°					

T= Temperature, t = time, RT = Room Temperature. Homogeneous subsets are divided in A or B.

Supplementary Table S7. Average measured Cq Values (n=2) with RT-qPCR using five cacao specific reference genes on 3% CTAB extracted RNA from six FD RT transported CCN cacao bean (sample 1 to 6).

**Sample**	**ACT-P**	**MDH**	**ACT-L**	**GADPH**	**RPL35**
1	26.2	25.93	27.37	25.27	25.69
2	27.56	25.62	27.79	25.19	25.47
3	28.11	27.32	28.80	26.56	27.02
4	27.80	27.14	29.24	26.76	27.39
5	29.97	28.42	29.49	28.02	28.07
6	28.21	27.04	29.05	26.91	26.63
**Average**	27.98	26.91	28.62	26.45	26.71
**SD**	1.22	1.01	0.85	1.074	1.00

2 Supplementary figures

Supplementary Figure S1. Different RNA Electropherograms resulting from the fragment analyzer™ (Advanced analytical technologies) ordered by shape quality values. Visual score legend: A = slightly degraded, B = partially degraded, S25 and S18 peaks visible, C = partially degraded, S25 and S18 peaks hardly visible with unreliable S25/S18 ratio, D = completely degraded, S25 and S18 peaks hardly visible with a lot of small fragments, E = completely degraded with unreliable S25/S18 ratios and F = failed sample. Light grey and dark grey peak indicate 28 and 25s rRNA, respectively.

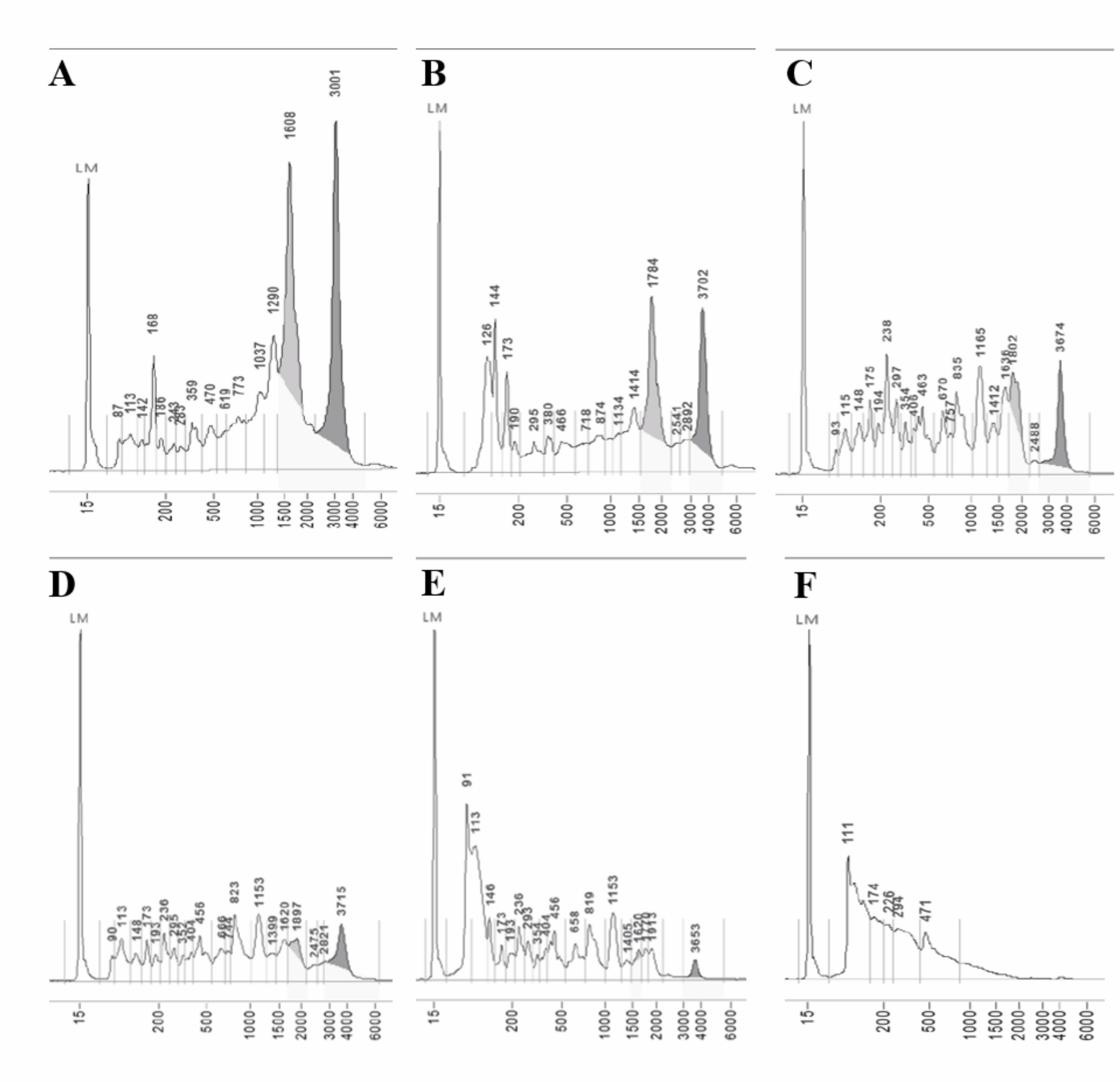

Supplement: Supplementary file 1 [file DataSheet_1.pdf]
